# Ecology and diversity of metazoan parasites infecting *Geophagus altifrons* (Cichliformes: Cichlidae) from the Amazon River system in northern Brazil

**DOI:** 10.1590/S1984-29612022014

**Published:** 2022-03-14

**Authors:** Ivanildo Amanajás Brito-Júnior, Marcos Sidney Brito Oliveira, Marcos Tavares-Dias

**Affiliations:** 1 Faculdade de Macapá – FAMA, Macapá, AP, Brasil; 2 Programa de Pós-graduação em Biodiversidade Tropical – PPGBio, Universidade Federal do Amapá – UNIFAP, Macapá, AP, Brasil; 3 Embrapa Amapá, Macapá, AP, Brasil

**Keywords:** Freshwater fish, aggregation, helminth, infection, Peixe de água doce, agregação, helminto, infecção

## Abstract

The aim of this study was to investigate the ecology and diversity of community and infracommunities of metazoan parasites *Geophagus altifrons* (Heckel, 1840) in Rio Jari, in the state of Amapá, in the eastern Amazon region. From the total of 31 fish examined, 90.3% were parasitized by one or more species, collecting a total number of 806 parasites. The parasites species identified were: *Sciadicleithrum geophagi*, *Posthodiplostomum* sp., *Procamallanus* (*Spirocamallanus*) *inopinatus*, *Raphidascaris* (*Sprentacaris*) sp., *Genarchella genarchella*, *Gorytocephalus spectabilis* and *Ergasilus xinguensis*. Most of the parasites showed an aggregate dispersion pattern. Brillouin diversity index, uniformity and species richness of parasites were low. The component community of parasites was constituted by ectoparasites and endoparasites, but with a predominance of ectoparasites. The body size of hosts had a low effect on the parasites infracommunities. This first eco-epidemiological study for *G. altifrons* reports these parasites in a host, for the first time, with the exception of *S. geophagi* and *E. xinguensis*.

## Resumo

O objetivo deste estudo foi investigar a ecologia e a diversidade da comunidade e infracomunidades de parasitos metazoários em *Geophagus altifrons* (Heckel, 1840), do Rio Jari, estado do Amapá, na região da Amazônia oriental. De um total de 31 peixes examinados, 90,3% estavam parasitados por uma ou mais espécies e 806 parasitos foram coletados. As espécies de parasitos identificadas foram: *Sciadicleithrum geophagi*, *Posthodiplostomum* sp., *Procamallanus* (*Spirocamallanus*) *inopinatus*, *Raphidascaris* (*Sprentacaris*) sp., *Genarchella genarchella*, *Gorytocephalus spectabilis* e *Ergasilus xinguensis*. A maioria dos parasitos apresentaram dispersão agregada. Foi baixo o índice de diversidade de Brillouin, uniformidade e riqueza de espécies de parasitos. A comunidade componente de parasitos foi constituída por ectoparasitos e endoparasitos, mas com predominância de ectoparasitos. O tamanho corporal dos hospedeiros teve baixo efeito sobre as infracomunidades dos parasitos. Este primeiro estudo ecoepidemiológico para *G. altifrons* registra pela primeira vez estes parasitos para este hospedeiro, exceto *S. geophagi* e *E. xinguensis*.

## Introduction

Species of Cichlidae are fish with innumerous species distributed particularly in fresh and brackish waters from the Central and South America and Africa; however, some species were introduced in other regions. *Geophagus altifrons* (Heckel, 1840), an ornamental cichlid from the Amazon River basin in Brazil with sedentary habits, is a benthopelagic and omnivorous fish that feeds on large-bodied benthonic organisms, terrestrial and aquatic insects, crustaceans and fish, algae, detritus, macrophytes, fruits and seeds, depending on the seasonal period. This fish that inhabits lakes and rivers have a maximum length of 27 cm, and its first maturation occurs with 15-17 cm (Soares et al., 2011; Hawlitschek et al., 2013; Camargo et al., 2015; Dary et al., 2017; Froese & Pauly, 2021).

For *G. altifrons*, *Ergasilus xinguensis* (Taborda, Paschoal & Luque, 2016) and *Sciadicleithrum geophagi* (Kritsky, Thatcher & Boeger, 1989) (Paschoal et al., 2016), are the only two species that have been described, due to the paucity of studies carried out. Despite their economic importance in the Amazon, studies focusing on the parasite fauna of *G. altifrons* have been not performed; hence, the ecology and diversity of parasites remain unknown. This species is not on the Red List of Threatened Species. Therefore, further studies on the ecology and diversity of the parasite in wild *G. altifrons* populations are need.

In wild fish populations, communities and infracommunities of parasites are the result of repeated additions and losses of parasite species during evolutionary history. Developing over time, the ecological and biological characteristics of hosts and parasites determine host colonization, consequently influencing parasite community richness and diversity (Poulin, 2004; Hoshino et al., 2014; Oliveira et al., 2017; Tavares-Dias et al., 2017a, b). The study of the factors influencing host-parasite interactions in wild fish populations has been gaining the interest of ecologists and parasitologists, resulting in an increase in the number of studies on this subject in the last decades.

Host age, size, diet, habitat, behavior, distribution and geographical range have been recognized as some of the factors influencing richness and diversity of parasite communities in wild fish populations (Poulin, 2004; Lagrue et al., 2011; Hoshino et al., 2014; Oliveira et al., 2017; Tavares-Dias et al., 2017 a, b; Pelegrini et al., 2021). Therefore, since the factors controlling infection levels, richness and diversity of parasite communities are diverse and can likely can vary spatially in wild fish populations, such factors need to be analyzed for different fish species, including *G. altifrons*. The aim of this study was to investigate the ecology and diversity of community and infracommunities of metazoan parasites in *G. altifrons* of a tributary from the Amazon River system in northern Brazil.

## Material and Methods

### Sampling area and fish collection

**Figure 1:**
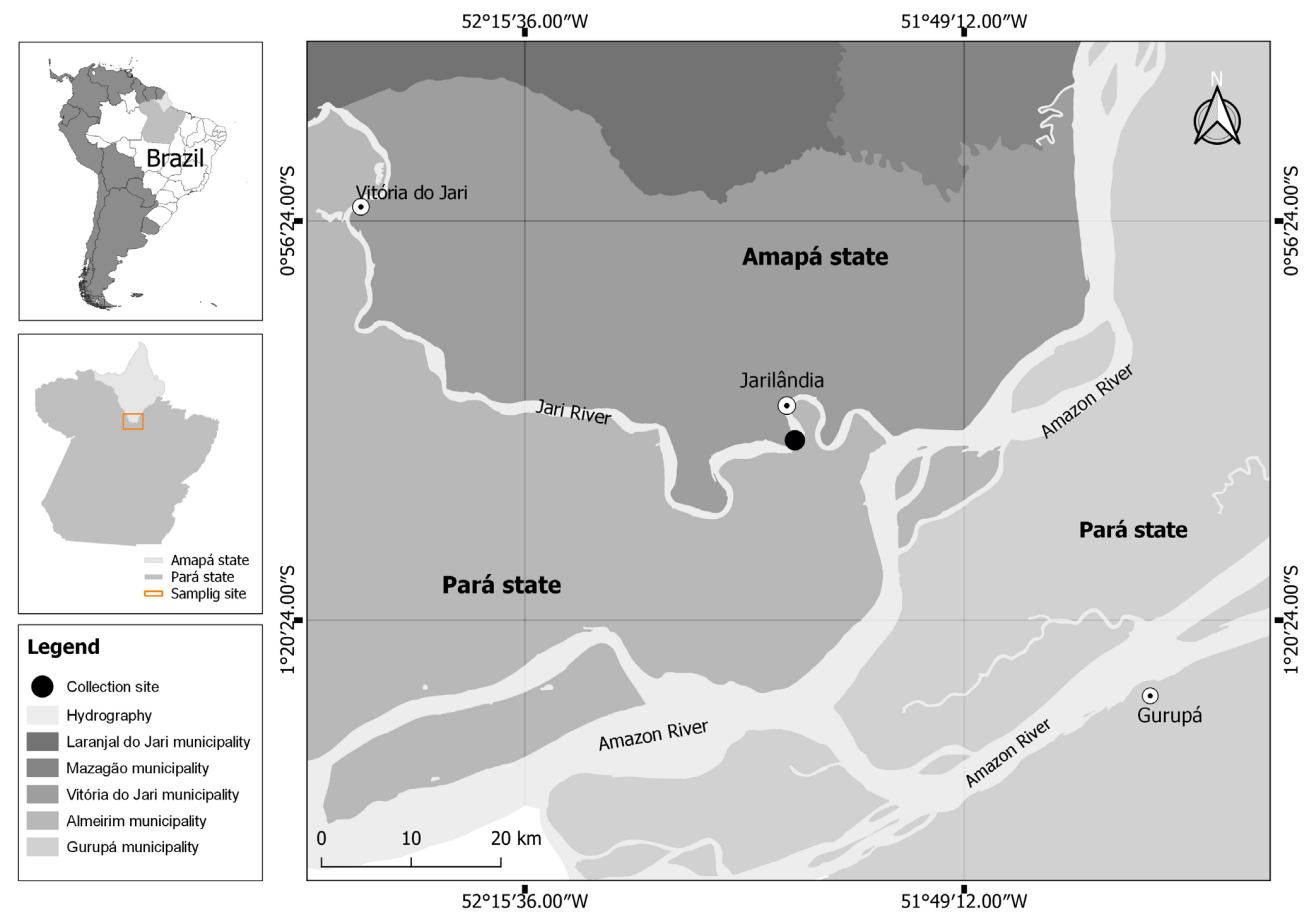
Collection site of *Geophagus altifrons* in Jari River, State of Amapá, northern Brazil.

Thirty-one specimens of *G. altifrons* (16.9 ± 2.7 cm and 108.6 ± 49.9 g) were collected in the Jari River basin, Jarilândia community, in the municipality of Vitória do Jari, Amapá state, Brazil (Figure 1), for parasitological analysis. Fish were captured using gill nets with 20 to 40 mm mesh.

The present study was carried out according to the recommendations and guidelines of the Brazilian College of Animal Experimentation (COBEA) and with authorization from the Ethics Committee on the Use of Animals of Embrapa Amapá (Protocol No 014 - CEUA/CPAFAP).

### Parasite collection and analysis procedures

Fish were weighed (g) and measured in total length (cm) and then necropsied for parasitological analysis. The mouth, operculum, gills, viscera and gastrointestinal tract were examined in each fish to collection of ectoparasites and endoparasites, using a stereomicroscope. The collection, fixation, conservation, counting and preparation of the parasites for identification followed the recommendations of Eiras et al. (2006). Parasites were identified according to Moravec (1998) and Thatcher (2006) and also in specialized papers

The ecological terms used were those recommended by Bush et al. (1997). Brillouin diversity index (*HB*), evenness (*E*) and species richness (Magurran, 2004) and dominance frequency (Rohde et al., 1995) were calculated to evaluate the community component of parasites using Diversity software (Pisces Conservation Ltd, UK). The dispersion index (ID) and discrepancy index (D) were calculated using the Quantitative Parasitology 3.0 software to detect the distribution pattern of parasite infracommunities (Rózsa et al., 2000) for species with a prevalence >10%. The significance of the ID was calculated using the *d*-statistic with Quantitative Parasitology 3.0 software, as well as the Poulin discrepancy index (D) (Ludwig et al., 1988). Spearman's correlation coefficient (*rs*) was used to investigate possible correlations of body length and weight of host with the richness of parasite species, Brillouin diversity index and parasite abundance (Zar, 2010).

## Results

In *G. altifrons*, a total of 806 parasites were collected such as *S. geophagi*, *Posthodiplostomum* sp., *Procamallanus* (*Spirocamallanus*) *inopinatus* (Travassos, Artigas & Pereira, 1928), *Raphidascaris* (*Sprentacaris*) sp., *Genarchella genarchella* (Travassos, Artigas & Pereira, 1928), *Gorytocephalus spectabilis* (Machado-Filho, 1956) and *E. xinguensis* (Table 1).

**Table 1:** Metazoan parasites in *Geophagus altifrons* from the Amazon River system in northern Brazil.

**Parasite species**	**P (%)**	**MI ± SD**	**MA ± SD**	**TNP**	**FD (%)**	**SI**
*Sciadicleithrum geophagi*	48.4	5.7 ± 5.0	2.8 ± 4.5	86	10.7	Gills
*Posthodiplostomum* sp.	83.9	1.0 ± 4.5	0.8 ± 2.5	25	3.1	Gills
*Genarchella genarchella*	16.1	1.8 ± 1.1	0.3 ± 0.8	9	1.0	Intestine
*Procamallanus* (S.) *inopinatus*	45.2	7.9 ± 12.0	3.6 ± 8.9	111	13.8	Intestine
*Raphidascaris* (*Sprentacaris*) sp. (larvae)	32.3	7.1 ± 11.5	2.3 ± 7.4	71	8.0	Intestine
*Gorytocephalus spectabilis*	12.9	1.5 ± 0.6	0.2 ± 0.6	6	0.7	Intestine
*Ergasilus xinguensis*	74.1	21.7 ± 16.6	16.1 ± 16.9	498	61.8	Gills

P: Prevalence, MI: Mean intensity, MA: Mean abundance, FD: Frequency of dominance, SI: Site of infection, TNP = total number of parasites.

Parasites showed an aggregate dispersion, except *G. genarchella* and *G. spectabilis* that showed a random dispersion (Table 2). From the total of 31 examined fish, prevalence of 90.3% was recorded, and the Brillouin diversity index showed that evenness and species richness were low (Table 3).

**Table 2:** Index of dispersion (ID), *d*-statistical (*d*) and discrepancy index (D) of parasite infracommunities in *Geophagus altifrons* from the Amazon River system in northern Brazil.

**Parasite species**	**ID**	***d***	**D**	**Type of dispersion**
*Sciadicleithrum geophagi*	2.55	4.19	0.64	Aggregate
*Posthodiplostomum* sp.	1.80	2.21	0.85	Aggregate
*Procamallanus* (*S.*) *inopinatus*	2.54	4.16	0.67	Aggregate
*Raphidascaris* (*Sprentacaris*) sp.	2.30	3.56	0.74	Aggregate
*Genarchella genarchella*	1.39	0.95	0.84	Random
*Gorytocephalus spectabilis*	1.52	1.37	0.86	Random
*Ergasilus xinguensis*	2.42	3.87	0.48	Aggregate

**Table 3:** Component community of metazoan parasites in in *Geophagus altifrons* from the Amazon River system in northern Brazil.

**Parameters**	**Values**
**All species of parasites**	
Number of hosts examined	31
Total prevalence (%) of parasites	90.3
Total number of parasites	806
Number species of parasites	7
Index of Brillouin diversity	0.32 ± 0.37
Evenness	0.18 ± 0.21
Species richness of parasites	1.71 ± 1.22
**Species of endoparasites**	
Number species of endoparasites	4
Percentage of endoparasites (%)	9.7
Species of endoparasites (larvae)	1
Species of endoparasites (adults)	3
**Species of ectoparasites**	
Number species of ectoparasites	3
Percentage of ectoparasites (%)	90.3
Species of ectoparasites (larvae)	0

**Figure 2:**
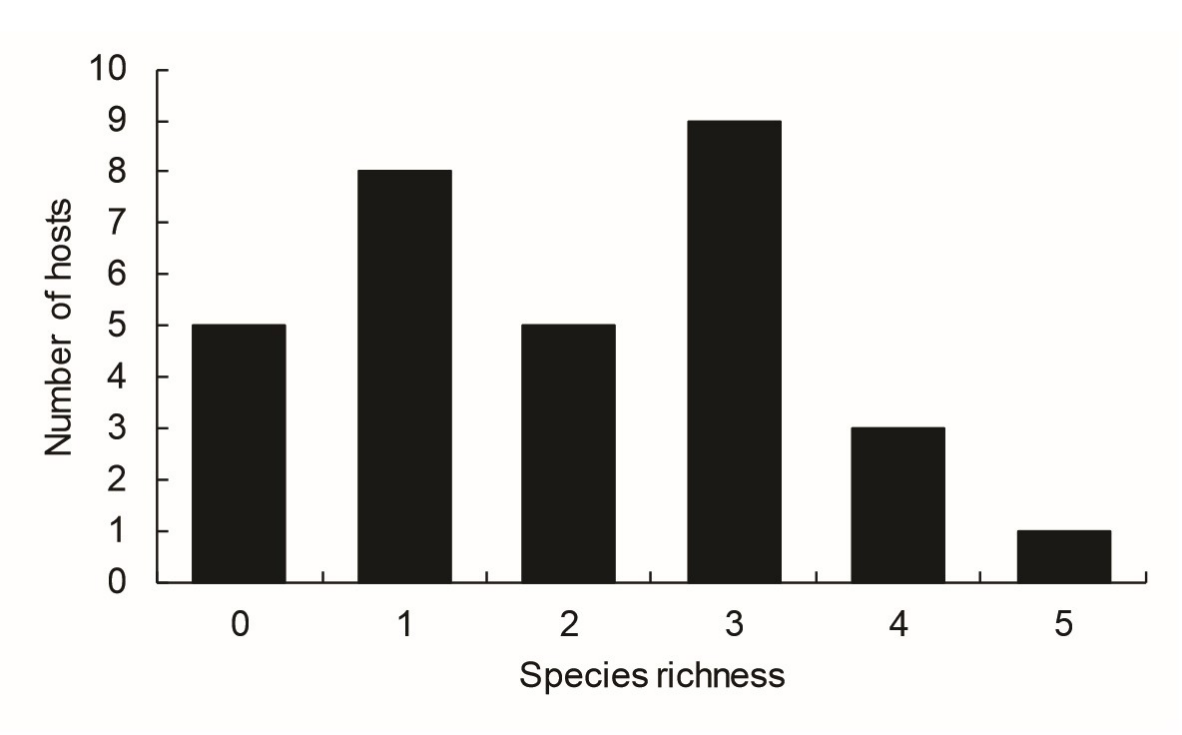
Species richness of metazoan parasites in *Geophagus altifrons* from the Amazon River system in northern Brazil.

There was a predominance of parasites infected by 2 to 4 species (Figure 2).

There was no significant correlation of the Brillouin diversity index (*rs* = 0.19, p = 0.29) and species richness (*rs* = 0.18, p= 0.31) with the length of hosts.

The abundance of *S. geophagi* (*rs* = 0.34, p = 0.06), abundance of *Posthodiplostomum* sp. (*rs* =0.08, p = 0.65), abundance of *Raphidascaris* (*Sprentacaris*) sp. (*rs* = 0.17, p = 0.36), abundance of *G. spectabilis* (*rs* = -0.03, p= 0.85) and abundance of *G. genarchella* (*rs* = -0.006, p = 0.97) showed no correlation with host length. The abundance of *E. xinguensis* (*rs* = 0.38, p = 0.03) presented a week positive correlation with the length of hosts, as well as the abundance of *P.* (*S.*) *inopinatus* (*rs* = 0.52, p = 0.002). The abundance of *G. spectabilis* (*rs* = -0.07, p= 0.70), abundance of *S. geophagi* (*rs* = 0.27, p = 0.15), abundance of *Posthodiplostomum* sp. (*rs* = 0.12, p = 0.53) and abundance of *Raphidascaris* (*Sprentacaris*) sp. (*rs* = 0.17, p = 0.37) showed no correlation with the host weight. The abundance of *E. xinguensis* (*rs* = 0.43, p = 0.01) and *P.* (*S.*) *inopinatus* (*rs* = 0.51, p= 0.003) presented a week positive correlation with the host weight.

## Discussion

Wild fish populations act as hosts for various taxa of parasites with varied life cycle strategies. Hence, the diversity and richness of parasite species depends on the host species and other factors related to the host and environment such as parasite life cycle, feeding habits and the reproductive stage of the host fish. Furthermore, they depend on the presence of intermediate hosts in the environment (Tavares-Dias et al., 2017a, b; Hoshino et al., 2014; Pelegrini et al., 2021). In *G. altifrons* from Jari River, the community of metazoan parasites was composed by seven species, those being: Monogenea, two Digenea, two Nematoda, one Acanthocephala and one Crustacea; parasites with aggregate or random dispersion pattern. The community of metazoan parasites in *Satanoperca jurupari* (Heckel, 1840) from Igarapé Fortaleza River was composed of eight species, those being: Monogenea, three Digenea, one Nematoda, one Acanthocephala and two Crustacea; all parasites with an aggregate dispersion (Tavares-Dias et al., 2017a). However, in *Cichlasoma bimaculatum* (Linnaeus, 1758) from the Igarapé Fortaleza River, the parasite community was composed of only three species, those being: one Monogenea, one Digenea and one Nematoda; parasites with aggregated dispersion (Tavares-Dias et al., 2017b). Therefore, such differences in the composition of the diversity and species richness of parasites are attributed to differences in host fish species, environment, age, size, diet, behavior, and distribution and geographical range.

Monogenea are common ectoparasites in freshwater fish populations from different ecosystems and their level of infection depends on environmental quality and habitat type (Ferreira-Sobrinho & Tavares-Dias, 2016; Tavares-Dias et al., 2017b). In the gills of *G. altifrons* the levels of infection by *S. geophagi* were lower than in *Geophagus camopiensis* (Pellegrin, 1903) (Ferreira-Sobrinho & Tavares-Dias, 2016). In the gills of *G. altifrons* there was also an infection by metacercariae of *Posthodiplostomum* sp., a digenean parasite that can be found in several freshwater fish around world (Tavares-Dias et al., 2017b). The life cycle of the *Posthodiplostomum* species involves two intermediate hosts, those being a fish species and a mollusk species, and a definitive host, which is a fish-eating bird (Ritossa et al., 2013; Tavares-Dias et al., 2017b). Therefore, *G. altifrons* is an intermediate host for metacercariae of *Posthodiplostomum* sp.

*Genarchella genarchella* occurred at low levels of infection in the intestine of *G. altifrons* from Jari River as reported for *Hemibrycon surinamensis* (Gery, 1962) from Igarapé Fortaleza River (Hoshino et al., 2014). This species of digenean has mollusk species and smaller fish as intermediate hosts, and large Characiformes and Siluriformes as definitive hosts (Martorelli, 1989; Lefebvre & Poulin, 2005). Therefore, *G. altifrons* was infected by this digenean through ingestion of infected mollusks or by direct contact with cercariae of *G. genarchella* in the environment.

*Procamallanus* (*S.*) *inopinatus* occurred at low levels of infection in *G. altifrons.* This nematode species, usually frequent in wild fish populations in Brazil, has fish as definitive hosts and in general has low levels of prevalence, intensity and abundance due to its complex life cycle (Neves et al., 2020). The abundance of *P.* (*S.*) *inopinatus* increased with the weight and length of the hosts. It can be assumed that larger host fish are ingesting more infective stages of this nematode than smaller hosts. In addition, in *G. altifrons* of the Jari River, larvae of *Raphidascaris* (*Sprentacaris*) sp. are at infection levels lower than those reported for *Geophagus proximus* (Castelnau, 1855) found in the Tapajós River (PA) (Oliveira et al., 2017). Species of *Raphidascaris* (*Sprentacaris*) have cladocerans as first intermediate hosts, smaller fish as secondary hosts and predatory fish as definitive hosts (Moravec, 1970, 1998). *Gorytocephalus spectabilis*, occurred at low levels of infection in *G. altifrons*, which is a definitive host for this acanthocephalan, with unknown life cycle.

Ergasilidae is one of the largest families of order Cyclopoida, and most species are found in freshwater fish. Just adult females have parasites in the gills, fins and nasal cavities of fish species (Taborda et al., 2016). *Ergasilus xinguensis* occurred at high levels of infestation in the gills of *G. altifrons* from the Jari River when compared to those reported for *Geophagus argyrostictus* (Kullander, 1991) and *G. altifrons* from Rio Xingu, in the state of Pará (Taborda et al., 2016). This is the second report of *E. xinguensis* for *G. altifrons*. Furthermore, abundance of *E. xinguensis* increased with the weight and length of hosts.

In conclusion, the component community of parasites in *G. altifrons* was composed by ecto- and endoparasite species with aggregate dispersion, but with a predominance of ectoparasites. This omnivorous fish occupies a lower position in the food web and is consumed by other larger fish species and fish-eating birds in the environment. The body size of hosts had a low influence on the parasites infracommunities. This first eco-epidemiological study of *G. altifrons* records, for the first time, these parasites for this host, except for *S. geophagi* and *E. xinguensis*.
